# Biological Prototype Acquisition Based on Biological Coupling in Bionic Design

**DOI:** 10.1155/2022/8458243

**Published:** 2022-10-15

**Authors:** Zhonghang Bai, Meijia Song, Xu Zhang, Jiahui Zhang

**Affiliations:** ^1^College of Architecture and Art Design, Hebei University of Technology, Tianjin 300130, China; ^2^National Technological Innovation Method and Tool Engineering Research Center, Tianjin 300401, China

## Abstract

Because the judgment basis in the process of biological prototype screening is highly subjective, and because it is difficult to generate a scheme when using multiple biological prototypes for bionic design, this work proposes a biological prototype retrieval and matching method for multibiological prototype bionic design. Using BioTRIZ in combination with biological coupling mechanism analysis, orthogonal analysis, and the calculation of the goodness value of the scheme, a multibiological prototype bionic design model is constructed. First, the biological prototype contradictions matrix is obtained by BioTRIZ. Then, a biological coupling mechanism analysis is carried out to calculate the goodness value of the auxiliary scheme to further evaluate the advantages and disadvantages of the biological prototype. The orthogonal analysis is then conducted to select the optimal biological prototype combination scheme. Finally, the best biological prototype combination scheme is transformed into the final design scheme according to the biological coupling mode prompts. According to this process, the innovative design of an automatic food threading machine was carried out, and an experiment was conducted for verification. The results demonstrate that the machine after bionic improvement could meet the design requirements, and the feasibility and effectiveness of the established design model were verified.

## 1. Introduction

After evolution and biological evolution, nature has the ability to provide human beings with design solutions that save energy and improve efficiency [1, 2]. Based on the research and transformation of natural phenomena and biological systems, the bionic design provides a development direction for designers to carry out innovative product design [3].

The screening of biological prototypes suitable for solving problems is a major problem in product innovation design via bionics. A substantial amount of research has been conducted on the acquisition of biological prototypes, and staged progress has been made. Some scholars have researched the construction of databases based on the interoperability of engineering and biological fields. For example, Vattam et al. [4] developed a DANE prototype system that uses the structure–behavior–function (SBF) model to capture the functions of biological systems. Goel et al. [5] constructed a structure-based DINE, a functional indexing knowledge base with a behavioral level. Nagel et al. [6] developed the online database AskNature via the use of functional representation and abstraction technology to obtain biological prototypes. Researchers from the University of Bath in the United Kingdom created a theory of inventive problem solving (TRIZ)-based biological effect knowledge base [7]. Chakrabarti [8] developed IDEA-INSPIRE software and constructed a system information database. Wilson and Rosen [9] proposed a systematic method based on reverse engineering and constructed a biological system ontology database. Moreover, a series of methods have been used to obtain biological prototypes that can solve engineering problems based on a large amount of data. For example, Shu [10] searched biological knowledge data and related biological phenomena via cross-domain terms with keywords in the natural language format. Vincent et al. [11] proposed the BioTRIZ theory of bionic design, and obtained biological prototypes based on the invention principles. Liu et al. [12] quickly obtained biological prototypes by constructing the mapping of FPBS and SATF. Cao et al. [13] proposed a function-oriented biological analogy method, and used function-driven databases such as AskNature to obtain biological prototypes. Based on the preceding literature review, it is more common to directly establish a model to obtain biological prototypes than to build a database. The former method can yield biological prototypes from a wide range of fields, and is therefore a more suitable method for obtaining biological prototypes for bionic design. However, most of the existing bionic designs are simple product designs for a single biological prototype. With the increasing complexity of products, it is difficult for the bionics of a single biological prototype to meet the application requirements. To date, some researchers have made preliminary attempts to study the bionic design method using multi-biological prototypes. For example, Chen et al. [14] obtained contradictory biological prototypes for bionic design from the perspective of multitechnology contradictions. Liu et al. [15] extracted and combined the parts of the multibiological effect model that complete the main functions to obtain technical solutions that meet the design requirements. Bai et al. [16] used TRIZ in the field of bionics (BioTRIZ) to obtain multiple pairs of contradictions, and extracted the main contradictions for green bionic design. The determination of how to retrieve biological prototypes has a profound theoretical research basis, but the screening and matching process of biological prototypes primarily depends on the subjective judgment of designers, and is too dependent on the degree of knowledge in related fields. Thus, it is necessary to establish a bionic design method that can integrate the advantages of multiple biological prototypes and is easy to implement.

Biological coupling is a method by which to analyze the general phenomena and laws existing in the biological world from the perspective of biology [17], and has been widely used in the field of bionics. For example, Xu et al. [18] introduced coupled bionics into the product modeling design process, and combined it with the topology analysis method to improve the efficiency and accuracy of product bionic design. Tian et al. [19] used solid coupling simulation technology to analyze the drag reduction characteristics of the surface layer material and the base bionic morphology of the morphology/material coupled bionic functional surface. Biological coupling theory holds that organisms combine or synergize multiple coupling elements to obtain an aggregate with at least one type of biological function via suitable coupling methods [17, 20–24]. In this work, biological prototypes are analyzed and disassembled via the biological coupling mechanism, and the basic components of the completed functions are determined to assist the calculation of the appropriateness of the biological prototypes, judge the advantages and disadvantages of the biological prototypes, and reduce the subjectivity of the selection of biological prototypes. Regarding the coupling mode for combinatorial bionics, the application of biological coupling breaks through the barriers between biology and technology based on the evaluation of biological prototypes and the mapping of biological prototypes to technical systems. Compared to a single bionic design, the bionic design based on the characteristics of multiple biological prototypes extracted from the biological coupling can better solve engineering problems.

In this work, BioTRIZ is used to obtain a large number of biological prototypes, and a biological prototype contradictions matrix is constructed. The biological prototypes are disassembled via mechanism analysis to calculate their appropriateness. Moreover, orthogonal analysis is carried out to consider the interaction between biological prototypes and bionics, and to further judge the biological prototypes. According to the advantages and disadvantages of the prototype combination, the best biological prototype combination is obtained. The bionic design is then carried out according to the prompts of the biological coupling mode.

## 2. Key Technologies

### 2.1. Construction of the Biological Prototype Matrix Based on BioTRIZ

TRIZ is an innovative method with numerous applications in engineering. It also has certain applications for the acquisition of biological prototypes in the field of bionics. For example, Liu et al. [25] combined physical and technical contradictions in TRIZ to obtain biological prototypes. Yusof et al. [26] combined TRIZ theory, morphological diagrams, and bionic concept design. However, Vincent et al. [11] found that the method of solving problems via bionics (BioTRIZ) is very different from the method of directly seeking solutions in the engineering field, namely TRIZ. Moreover, the biological prototype solutions sought by TRIZ are more complex than those sought by BioTRIZ, and their applicability to the product is low [11, 27]. Based on the TRIZ contradictions, BioTRIZ seeks solutions in the biological world that can connect engineering and biology, and simplifies the 39 × 39 PRIZM contradiction matrix as a 6 × 6 matrix, which is a win–win approach to ecological innovation [28]. For example, Jiji et al. [29] proposed an innovative design process model based on BioTRIZ according to the principle of relation-mapping-inversion (RMI). Salmaan et al. [30] used BioTRIZ to design a roof imitating a honeycomb structure to reduce thermal radiation. Regarding other applications of BioTRIZ, Bai et al. [16] combined BioTRIZ with green factors to achieve an innovative design. In the present work, a BioTRIZ-driven approach to finding biological prototypes is applied to rapidly obtain a large number of biological prototypes that can solve design problems. In view of the problems existing in the current application of BioTRIZ, the following application improvements are proposed.

1. In the case of multiple contradictions in the products that need to be innovatively designed, the products are divided into functional areas, and multiple pairs of contradictions are obtained to construct a biological prototype contradiction matrix.
2. Considering that the biological prototype search is currently limited to the biological prototype ontology database created by Vincent and others, the search can be extended via functions (e.g., biological incentive websites such as AskNature), and can also be carried out with major search engines.

**Figure 1:**
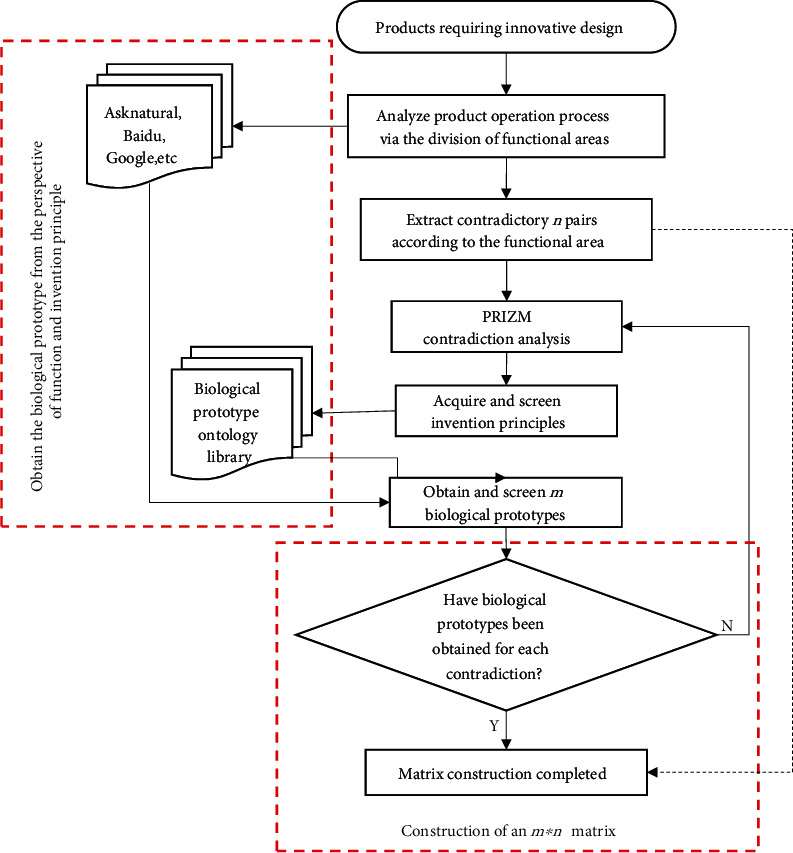
The flowchart of the construction of the biological prototype matrix based on BioTRIZ.

The current research and application of BioTRIZ are transformed, and the flowchart of the construction of the biological prototype matrix based on BioTRIZ is presented in Figure 1.

### 2.2. Evaluation of Biological Prototypes Based on Biological Coupling Mechanism Analysis

After obtaining the biological prototype matrix via BioTRIZ, it is necessary to screen the biological prototypes to obtain the optimal biological prototype for bionic design. There are differences in the evaluation methods of biological prototypes used in bionics. For example, Liu et al. [31] used topology to evaluate and select biological prototypes from the four levels of function, action, strategy, and structural similarity calculation. Jia et al. [32] analyzed the similarity of biological prototypes via the system function description module and the system similarity module. Cao et al. [13] used the TFN computing algorithm to evaluate the suitability of the retrieved biological prototypes. Hou et al. [33] adopted an analytical and biofunctional combinatorial model to screen biological prototypes. At present, the evaluation of biological prototypes is mostly considered from the perspective of engineering, and there is a lack of evaluation from the perspective of biology. Biological coupling mechanism analysis is an analysis method in which the principle of extension is applied to analyze the biological coupling mode, after which a multicoupling model is established. It provides a method to explore multi-coupling bionics [20–22]. Via the mechanism analysis of the biological prototype, the coupling elements that contribute to the function of the prototype are obtained to judge its advantages and disadvantages.

In this study, a biological prototype evaluation method based on biological coupling mechanism analysis is proposed. Biological archetypes are evaluated according to the various levels of coupling elements that contribute to biological functions. This method is divided into two parts, namely the disassembly analysis of biological prototypes and the calculation of the goodness value of the scheme.

#### 2.2.1. Disassembly Analysis of Biological Prototypes

**Figure 2:**
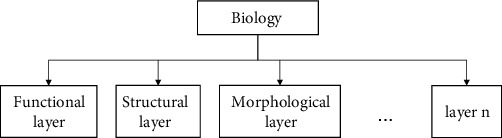
The multivariate coupling model.

The functions of organisms are formed by the contribution of each coupling element to different degrees [19–21]. The coupling element is the basic unit of biological coupling, and has different characteristics. Biological coupling elements are diverse, for example, material, structure, morphology, properties (such as flexibility, lubricity), behavior, etc. The biological prototype is disassembled according to the biological coupling mechanism analysis, and the expression of the multivariate coupling model after disassembly is presented in Figure 2.

#### 2.2.2. Calculation of the Goodness Value of the Scheme

**Figure 3:**
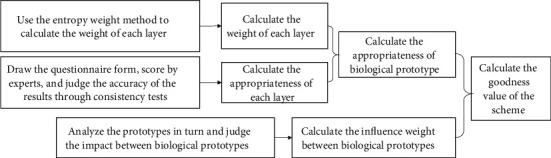
The scheme evaluation model.

The evaluation of multiple biomimetic schemes requires the consideration of not only the advantages and disadvantages of biological prototypes but also the mutual influence of biological prototypes [14]. Therefore, the calculation of the goodness value of the scheme is divided into two parts, namely the calculation of the appropriateness of biological prototypes, and the calculation of the influence weight between biological prototypes. The evaluation model is shown in Figure 3.

1. Calculation of the appropriateness of biological prototypes

The suitability of biological prototypes is the degree of suitability between the biological prototypes and product models that can perform corresponding functions [13].

(1) *Appropriateness calculation of each level:* A scoring table is created to judge the degree of realization of the target function in the technical system at each level differentiated by the coupling element. The prototypes are judged and scored by relevant experts (5: very high degree of achievement, 4: high degree of achievement, 3: average degree of realization, 2: low degree of realization, 1: very low degree of realization), and the expert scoring results are collected for data analysis, and Kendall consistency tests are conducted to avoid the error of expert judgment. Then, the appropriateness value of each biological prototype at each level is obtained.

(2) *Weight calculation:* The entropy weight method is used to calculate the weight of the appropriateness of each level.

First, the data are normalized, the positive index is calculated by equation (1), and the negative index is calculated by equation (2):

(1) $$\begin{array}{c} Y_{ij}=\frac{X_{ij}-\min\left(X_{i}\right)}{\max\left(X_{i}\right)-\min\left(X_{i}\right)},\left(i=1,2,3,...,n;j=1,2,3,...,m\right), \end{array}$$

(2) $$\begin{array}{c} Y_{ij}=\frac{\min\left(X_{i}\right)-X_{ij}}{\max\left(X_{i}\right)-\min\left(X_{i}\right)},\left(i=1,2,3,...,n;j=1,2,3,...,m\right). \end{array}$$

Then, the entropy of index (column) *j* is calculated as follows:

(3) $$\begin{array}{c} {Ε}_{i}=-\frac{1}{\ln n}\overset{n}{\underset{i=1}{\sum }}\rho_{ij}\ln\rho_{ij}, \end{array}$$

where

(4) $$\begin{array}{c} \rho_{ij}=Y_{ij}/\overset{n}{\underset{i=1}{\sum }}Y_{ij};\text{when }\rho_{ij}=0,\text{define}\underset{\rho_{ij}⟶0}{\lim}\rho_{ij}\ln\rho_{ij}=0. \end{array}$$

Finally, the weight of index (column) *j* is obtained as follows:

(5) $$\begin{array}{c} \omega_{i}=d_{i}/\left(k-\overset{n}{\underset{i=1}{\sum }}d_{i}\right), \end{array}$$

where

(6) $$\begin{array}{c} d_{i}=1-{Ε}_{i}. \end{array}$$

The appropriateness of each level and its weight are calculated according to the preceding steps, after which the appropriateness SIM value of the biological prototype can be obtained. The calculation is as follows:

(7) $$\begin{array}{c} \text{SIM}=\underset{i=1}{\sum }\omega_{i}\times \gamma_{i}, \end{array}$$

where *ω_i_* is the appropriateness of each level, and *γ_i_* is the appropriateness weight of each level.

(2) Calculation of the influence values between biological prototypes

To analyze the influence of biological prototypes, the bionic scheme of the bionic part of the biological prototype is first analyzed to determine whether the changes after bionics have mutual influences, such as contradictions between structures, contradictions in shape and size, contradictions in environmental conditions, etc. According to the degree of influence, the biological prototype is given a weight *ω*_*j*_ (0, influential and 1, no impact).

The results of these two methods are introduced into the scheme evaluation equation to calculate the final score of the multibiological prototype combination scheme:

(8) $$\begin{array}{c} Q_{\kappa }=\underset{j=1}{\sum }\omega_{j}{\text{SIM}}_{j}, \end{array}$$

where *ω*_*j*_ is the influence weight between biological prototypes, SIM_*j*_ is the appropriateness of the biological prototypes, and *κ* is the number of combinations.

### 2.3. Selection of the Optimal Biological Prototype Combination Based on Orthogonal Analysis

Orthogonal analysis is an analytical method by which to study multiple factors and multiple levels in which some representative combinations are selected from all combinations for comparison based on orthogonality [34]. In orthogonal analysis, representative combinations are extracted via a fractional factorial design, and combinations that are not considered are removed. It is mainly based on the following: (1) the principle of effect sparsity: the system or process is usually dominated by several main effects and low-cost interactions; (2) the projection effect: in the linear generation, one vector is projected on the other vector as a point, and at this time, the two vectors have no influence on each other. Orthogonal analysis has a large number of applications as a universal method for selecting the optimal combination. For example, Bai et al. [35] used orthogonal analysis to assist in judging the degree of correlation between affordability factors and product modeling factors. Feng and Qin [36] analyzed the characteristics of the front longitudinal beams of automobiles with different cross-sections via cross-section tests. Hu [37] applied preference scores to orthogonal analysis, and demonstrated that nondeterministic data can also be applied to orthogonal analysis. Song and Zou [38] combined subjective and objective data measurement via orthogonal analysis to analyze the factors that influence the back pressure comfort of military training uniforms for college students. The preceding review of the application of orthogonal analysis shows that it is suitable for relatively complex product design, and can be used to analyze the problems caused by multiple factors and to select the best level of factors.

**Figure 4:**
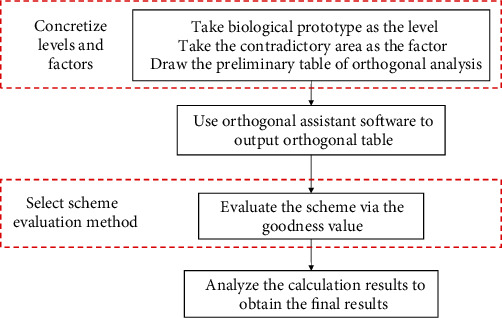
The flowchart of the selection of the optimal biological prototype combination via orthogonal analysis.

When there are too many contradictory regions and biological prototypes, too many combinations can be formed. In this work, the orthogonal analysis method is used to screen out representative biological prototype combinations for analysis. For objects with chaotic influence factors, orthogonal analysis can be conducted based on experimental data [36], while for product design, the influence of factors is predictable and computable [14, 37], and the calculation of the goodness value directly outputs the results of each scheme. The application transformation of orthogonal analysis in this study is shown in Figure 4. The optimal biological prototype combination is obtained via the data analysis of the goodness value of the combination provided by the orthogonal table.

The steps of orthogonal analysis combined with the goodness value of the scheme to select the optimal biological prototype combination are as follows. First, the initial data factor number (contradiction area) and level number (biological prototype corresponding to the contradiction) are input into orthogonal analysis software, and an orthogonal table is then obtained. The goodness value of the scheme is then calculated according to the prompt of the orthogonal table, the results are analyzed intuitively, and the optimal multibiological prototype combination scheme is selected.

## 3. Process

**Figure 5:**
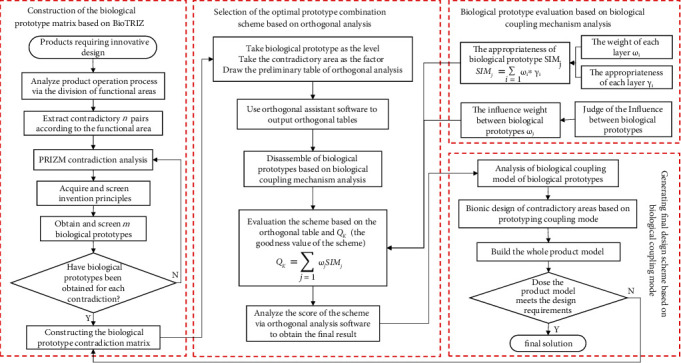
The biomimetic product design process is based on biological coupling.

The bionic product design process of BioTRIZ-driven multibiological prototype solution is presented in Figure 5.

### 3.1. Construction of the Biological Prototype Matrix Based on BioTRIZ

1. First, the whole product must be innovatively designed. According to the product operation process, the product is divided into different functional areas by functional decomposition, and the contradiction analysis is carried out according to the functional areas to obtain multiple pairs of contradictions.
2. According to the deterioration and improvement domains in the six operation domains corresponding to the contradiction, the PRIZM of BioTRIZ is searched to obtain the corresponding invention principle.
3. The product contradictions are analyzed via the principle of preliminary invention screening.
4. After obtaining the screened invention principle, the biological prototypes can be obtained in the biological prototype ontology library created by Vincent, Ask Natural, and other biological incentive websites, and can also be searched for via major search engines. When obtaining the biological prototypes, the search scope is expanded to obtain more biological prototypes.
5. After obtaining the biological prototypes, they are preliminarily screened to remove those that would be difficult to improve via bionics.
6. This step is completed after each contradiction yields the biological prototype. If there is a contradiction that does not yield the invention principle, the biological prototypes are researched based on the analysis of the contradictions.
7. According to the obtained contradictory regions and their corresponding biological prototypes, the biological prototype matrix can be obtained.

### 3.2. Biological Prototype Evaluation Based on Biological Coupling Mechanism Analysis

1. After obtaining each biological prototype, it is disassembled via biological coupling mechanism analysis.
2. The appropriateness of the biological prototype is calculated.
3. The scheme priority value after the combination of biological prototypes is calculated.

### 3.3. Selection of the Optimal Biological Prototype Combination Scheme Based on Orthogonal Analysis

1. The matrix, for which the contradictory functional region is the horizontal row and the biological prototype is the vertical column, is input into orthogonal analysis software to obtain the orthogonal table of *Lk*(*mn*) (where *k* is the number of experimental groups, i.e., the number of multi-biomimetic combination schemes, *m* is a level number, i.e., the number of conflicting functional areas, and *n* is the number of factors, namely the number of biological prototypes).
2. The bionic combination to be calculated is listed in the form of an orthogonal table. According to the *k* groups of bionic schemes suggested by the orthogonal table, the optimal value of the scheme is input and the data analysis is carried out. According to the results, the best multibiological prototype bionic combination is selected.

### 3.4. Generating the Final Design Scheme Based on the Biological Coupling Mode

1. In view of the functional areas that produce contradictions, the innovative design of the corresponding functional areas is carried out according to the corresponding biological prototypes.
2. A reasonable coupling mode is selected for the connection between each coupling element, the coupling of the bionic design of the biological prototype combination to the whole product is realized, and the final product design is completed.
3. The product can meet the design requirements of the end process. If there remain defects, the product can be transferred to the BioTRIZ driver to carry out further steps to continue the analysis.

## 4. Experimental Verification

**Figure 6:**
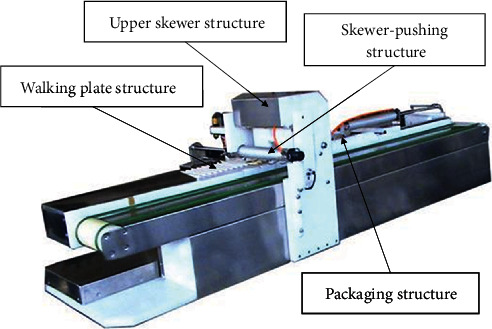
An automatic threading machine.

In the food processing industry, threading and barbecuing have basically achieved mechanical automation, but communication between the two is difficult. The gear skewers can be used for automatic barbecue machines, but they cannot be used for automatic threading machines. However, the ordinary skewer suitable for automatic threading machines cannot be applied to barbecue machines, and the efficiency of barbecuing cannot be maximized. In this study, according to the existing threading machine, such as that illustrated in Figure 6, a food stringer that can be applied to gear skewers is designed.

### 4.1. Construction of the Biological Prototype Matrix Based on BioTRIZ

#### 4.1.1. Analysis of the Current Situation and Demand

Determining how to improve the overall efficiency is a problem that must be considered in this innovative design. To solve this problem, the stringer, gear skewer, and barbecue machine can be improved. This example is highlighted by the design of the stringer, which can be applied to the gear skewer.

#### 4.1.2. Division of Functional Areas

First, the process-based functional area decomposition of the stringer is carried out. An ordinary threading machine is composed of a walking board and several components for pressing, preparing, pushing, and collecting. Among them, the structure related to the gear skewer cannot be used in the upper skewer structure (which prepares the skewer), the skewer-pushing structure (which pushes the skewer into the food), the walking plate (which movies the die plate, thus moving the food in the direction of the skewer), or the collection structure (which collects and sorts the food).

#### 4.1.3. Obtaining Contradictions

1. The upper skewer structure must reasonably distribute the skewers so that the later skewer-pushing structure can successfully complete the skewer-pushing function. To allow the upper skewer shaft to place the gear skewers, the placement groove can be expanded, but it will be difficult for the expanded skewers to move along a straight line; this can easily cause broken skewers. Thus, a contradiction of BioTRIZ is obtained, that is, the structure is improved but the material has deteriorated.
2. The skewer-pushing structure pushes the skewer into the food. At present, there is no improvement scheme, and the skewer can only be manually pierced to obtain the contradiction of BioTRIZ, that is, the structure is improved but the timeliness has deteriorated.
3. The structure of the walking plate can be improved by the shape of the die plate. The change of the shape of the skewer does not affect the movement of the die plate. The structure of the walking plate is changed to the die structure. The previous method in which only the groove of the die plate was changed is not suitable for the gear skewer or will cause the die plate to clamp the skewer gear, which will then be difficult to quickly remove. A contradiction of BioTRIZ is therefore obtained, that is, the structure is improved but the structure has also deteriorated.
4. Regarding the packaging structure, the finished products are collected one by one either by hand or by other machine structures. Thus, the contradiction of BioTRIZ is that the structure is improved, but the energy efficiency has deteriorated.

#### 4.1.4. Determine the Invention Principle

The PRIZM was obtained to determine the invention principle according to the contradictions, and the results are reported in Table 1.

#### 4.1.5. Obtain the Biological Prototypes

The biological prototypes were obtained from ontology biobanks developed by Vincent and biological incentive websites such as Ask Natural according to the invention principle and were supplemented with a browser search. The invention principles corresponding to biological prototypes are reported in Table 2.

#### 4.1.6. Drawing the Multibiological Prototype Matrix

The biological prototypes were screened to remove those which would be difficult or unable to create via bionics. The resulting matrix is presented in Table 3.

### 4.2. Selection of the Optimal Biological Prototype Combination Based on Orthogonal Analysis

#### 4.2.1. Establishing the Orthogonal Table

The L16(45) orthogonal table was obtained by inputting the information in Table 3 into orthogonal analysis software at the biological prototype level with the contradictory area as the factor, and 16 groups of schemes were obtained.

#### 4.2.2. Dismantling the Biological Prototypes

The screened biological prototypes were dismantled according to biological coupling mechanism analysis.

#### 4.2.3. Calculation of Scheme Optimization

1. Calculation of the appropriateness of the biological prototypes

A total of 23 professionals were invited to score the prototypes, and 22 valid scores were collected. The data were analyzed, and Kendall consistency tests were carried out. The results revealed strong consistency with values between 0.6 and 0.8, and the data are reported in Table 4.

According to the specific score of the appropriateness of each level, the appropriateness weight of each level was calculated by the entropy weight method. The results of the weighted biological prototype fitness *Qj* calculated by equation (7) are exhibited in Table 5.

(2) Calculation of the influence weights among biological prototypes

**Figure 7:**
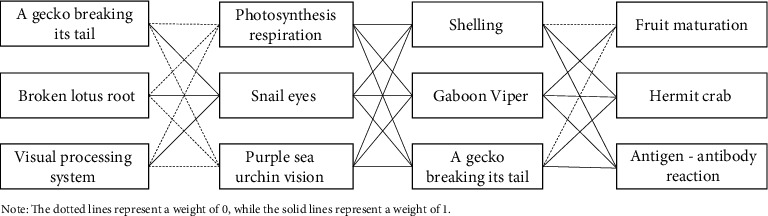
The analysis of the impacts between biological prototypes.

There are four conflicting components of the product analyzed. The upper skewer structure is connected to the skewer-pushing structure, the skewer-pushing structure is connected to the walking plate structure, and the walking plate structure is connected to the packaging structure, which will affect each other. The analysis reveals the influences between the following biological prototypes, as presented in Figure 7.

1. When “a gecko breaking its tail” is adopted for the upper skewer structure, the “photosynthesis respiration” and “purple sea urchin vision” of the skewer-pushing structure will be affected, resulting in the failure of the skewer-pushing structure; thus, the weight is 0.
2. When “a broken lotus root” is adopted for the upper skewer structure, the “photosynthesis respiration” and “purple sea urchin vision” of the skewer-pushing structure will be affected, resulting in the failure of the skewer-pushing structure; thus, the weight is 0.
3. When the “visual processing system” is adopted for the upper skewer structure, the “photosynthesis respiration” and “purple sea urchin vision” of the skewer-pushing structure will be affected, resulting in the failure of the skewer-pushing structure; thus, the weight is 0.
4. When “shelling” is adopted for the walking plate structure, the “fruit maturation” of the packaging structure will be affected, and the use efficiency will be low; thus, the weight is 0.
5. When “a gecko breaking its tail” is adopted for the walking plate structure, the “fruit maturation” of the packaging structure will be affected, and the use efficiency will be low; thus, the weight is 0.

In summary, according to the calculation of each combination score via eqution (8), the results were input into orthogonal analysis software for data analysis. The results are presented in Table 6.

The mean values of each structure in Table 6 were analyzed. To achieve the optimal overall design, the biological prototype with the maximum mean values of each structure was selected for the bionic design. The best effect was found to be achieved via the combination of A3, B2, C2, and D2.

### 4.3. Generating the Final Design Scheme Based on the Biological Coupling Mode

According to the results of orthogonal analysis, the best bionic combination was found to be obtained when the bionic reference for the upper structure is “the visual processing system”, the bionic reference for the skewer-pushing structure is “snail eyes”, the bionic reference for the walking plate structure is the “Gaboon viper”, and the bionic reference for the packaging structure is the “hermit crab.” The mapping method from the biological prototype to the mechanical structure is mainly divided into two parts: biological coupling disassembly and mechanical function realization. First, disassemble the obtained biological prototype through the coupling principle realized by the function of biological prototype, and further decompose it into functional, structural, morphological, or characteristic components; then look for mechanical components that can realize the functions of each component; finally, use Couplings between biological prototype components combine mechanical components. In this way, the biomimetic of the biological prototype is finally realized.

#### 4.3.1. The Upper Skewer Structure

**Figure 8:**
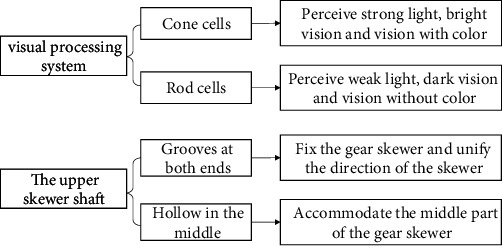
The schematic diagram of the biotechnology system transformation of the upper skewer structure.

**Figure 9:**
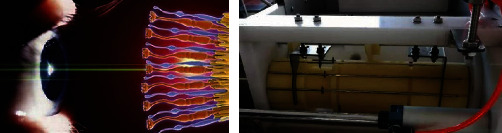
The biological prototype of the upper skewer structure.

The bionics of the upper skewer structure is akin to the human visual system. Cone cells and rod cells act as photoreceptors on the retina. Rod cells mainly perceive weak light, dark vision, and vision without color, and cone cells mainly perceive strong light, bright vision, and vision with color. Combining with the tips of the invention principle obtained above, there can be two different functional areas on the same object, corresponding to the upper string axis, and can also be divided into two areas. The two ends of the shaft are set with thin grooves, and the middle part connects the grooves so that it can accommodate the irregular part of the gear skewer. The schematic diagram of the biotechnology system transformation is presented in Figure 8, and the final structure is displayed in Figure 9.

#### 4.3.2. The Skewer-Pushing Structure

**Figure 10:**
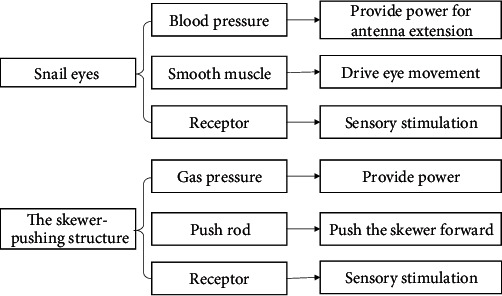
The schematic diagram of the biotechnology system transformation of the skewer-pushing structure.

**Figure 11:**
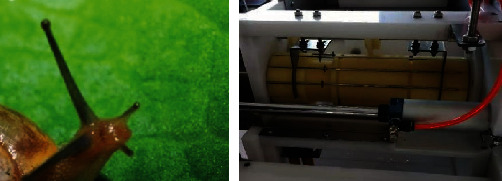
The biological prototype of the skewer-pushing structure.

The bionic effect of the skewer-pushing structure is akin to snail eyes. The eyes of a snail are located on its antennae, and the existing positions of the antennae include diastolic extension outside the body and systolic retraction inside the body. Antennal contraction is described as follows: when the receptors on the antennae are stimulated, the smooth muscle can contract through the reflex arc. Tentacle extension relies on blood pressure, via which the smooth muscle is extended. The biological prototype suggests that the same object can be transformed into different positions, and can adopt the contraction structure and provide power via pressure. The specific application is that the air cylinder pushes the skewer to produce displacement. The schematic diagram of the biotechnology system transformation is presented in Figure 10 below, and the final structure is displayed in Figure 11.

#### 4.3.3. The Walking Plate Structure

**Figure 12:**
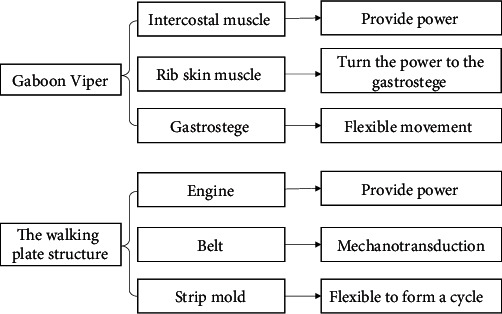
The schematic diagram of the biotechnology system transformation of the walking plate structure.

**Figure 13:**
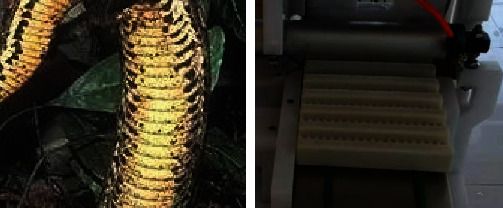
The biological prototype of the walking plate structure.

The bionics of the walking plate structure are similar to those of the Gaboon viper. Unlike common snakes, the Gaboon viper moves along a straight line, which is due to the crawler movement of its abdomen. The snake has no sternum, and its ribs can move back and forth freely via the contraction and relaxation of intercostal muscles. There are costocutaneous muscles between the ribs and gastrostege, and the position and state of the gastrostege change to drive the snake forward. The biological prototype prompted the division of the mold plate into blocks connected to the belt to move forward. The schematic diagram of the biotechnology system transformation is shown in Figure 12, and the final structure is displayed in Figure 13.

#### 4.3.4. The Packaging Structure

**Figure 14:**
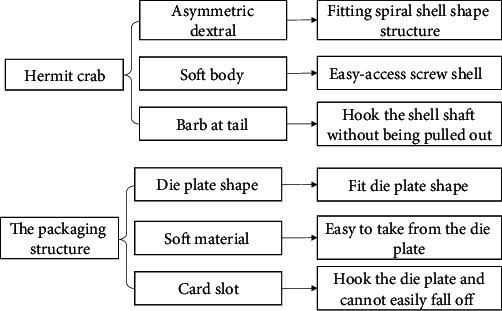
The schematic diagram of the biotechnology system transformation of the packaging plate structure.

**Figure 15:**
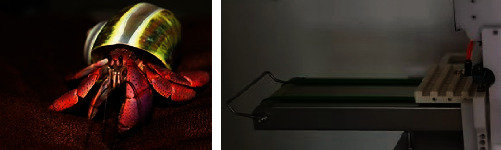
The biological prototype of the packaging structure.

The bionics of the packaging structure are similar to those of the hermit crab. The abdomen of the land hermit crab is asymmetric dextral (most marine spiral shells are also dextral), the body is soft, the surface is not covered by the shell (the crab can easily enter and exit the shell), and barbs are attached to the tail of the abdomen (tail joints and tail limbs), which can hook the shell shaft without being pulled out by a predator. According to the biological prototype, the packaging template (blister disk) was directly covered on the mold plate, and the bionic structure of the hermit crab was adopted. Hooks were designed at the tail to prevent the mold plate from falling. When the mold plate reached the hook, the plastic film on the mold plate with the rows of meat skewers was removed, and the skewers were directly packed in rows to avoid removing the meat skewers one by one. The schematic diagram of the biotechnology system transformation is shown in Figure 14, and the final structure is displayed in Figure 15.

#### 4.3.5. Scheme Evaluation

**Figure 16:**
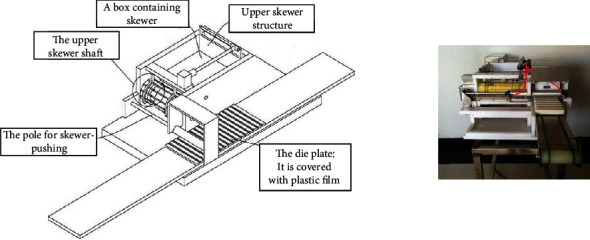
The gear piercing threading machine.

Based on the bionic transformation of each part, the final product output is exhibited in Figure 16 (patent number of the gear skewer and threading machine: cn21254503u). After the innovative design, the automatic threading machine can be applied to the gear skewer and threading. Moreover, the packaging part has been upgraded, which greatly reduces the use of human and material resources. The comparison before and after the improvement of the scheme is presented in Table 7.

## 5. Conclusion

Via the combined use of BioTRIZ, orthogonal analysis, biological coupling, and other theoretical methods, the problem of obtaining multiple biological prototypes caused by the aggravation of product complexity has been solved, and the objectivity of bionic design has been improved. However, at present, the influence of subjectivity on the research in this field cannot be completely avoided, and further improvements will be made in follow-up research.

Aiming at the multicontradiction problem in BioTRIZ, a biological prototype contradiction matrix based on BioTRIZ was proposed. The possible solutions to each contradiction are listed one by one, after which the optimal combination scheme is determined to solve the problem, thereby providing a new solution for the multicontradiction problem in BioTRIZ. In view of the current situation of biological prototype evaluation from the perspective of technical systems, the advantages and disadvantages of biological prototypes are compared from the perspective of biology. Biological coupling mechanism analysis and the appropriateness of biological prototypes are used to evaluate and screen biological prototypes, which provides a more reliable evaluation standard. By organically combining orthogonal analysis and the calculation of the scheme optimization value, the selection method of the optimal bionic schemes for multiple biological prototypes is innovatively developed, which effectively reduces the number of bionic schemes to be analyzed. The biological coupling mode is used to prompt the bionic design, and a new method for the application of bionic design biological prototype is ultimately provided.

## Figures and Tables

**Table 1:** The correspondence between the structure, contradiction, and invention principle.

Structure	Contradiction	Invention principle
Upper skewer structure	Improved structure, deteriorated material	1, 15, 19
Skewer-pushing structure	Improved structure, deteriorated time	1, 2, 4
Walking plate structure	Improved structure, deteriorated structure	1, 15, 19, 34
Packaging structure	Improved structure, deteriorated the energy	1, 3, 5, 25, 40

**Table 2:** The invention principles corresponding to the structures and biological prototypes.

	Invention principle	Biological prototypes
Upper skewer structure	1 Segmentation	A gecko breaking its tail; a cicada shedding its shell; a broken lotus root.
	15 Dynamicity	The visual processing system; rock ants nesting.
	19 Periodic action	Insects circulating nutrients; clownfish avoiding sea anemone thorns.
Skewer-pushing structure	1 Segmentation	A gecko breaking its tail; photosynthesis respiration.
	2 Extraction	Fireflies glowing; snail eyes; purple sea urchin vision.
	4 Asymmetry	Passive ventilation in black-tailed prairie rat caves.
Walking plate structure	1 Segmentation	A gecko breaking its tail.
	15 Dynamicity	Frog predation; spiral flow.
	19 Periodic action	Gaboon viper.
	34 Rejecting and regenerating parts	Shelling; molting.
Packaging structure	1 Segmentation	A gecko breaking its tail.
	3 Local condition	The visual processing system; shark skin.
	5 Consolidation	Antigen-antibody reaction; hermit crab.
	25 Self-service	Kangaroo pouches; auditory feedback; bat positioning; fruit maturation.
	40 Composite materials	Hedgehog spines...

Note: Some biological examples in this table were derived from the ontology biological instance database developed by Vincent and the online database AskNature (http://www.asknature.org), and their respective information was sourced from web search engines such as Wikipedia.

**Table 3:** The preliminary orthogonal analysis.

	A: upper skewer structure	B: skewer-pushing structure	C: walking plate structure	D: packaging structure
1	A gecko breaking its tail	Photosynthesis respiration	Shelling	Fruit maturation
2	A broken lotus root	Snail eyes	Gaboon viper	Hermit crab
3	The visual processing system	Purple Sea urchin vision	A gecko breaking its tail	Antigen-antibody reaction

Note: although the dimensions of this table are 4 × 3, the number of biological prototypes does not need to be the same in practical application.

**Table 4:** The data collation of the appropriateness of each layer.

Mean value	Functional layer	Structural layer	Material layer	Significant layer	Behavior layer
A1	0.60	0.23	—	0.09	0.08
A2	0.24	0.13	0.45	0.19	—
A3	0.14	0.09	—	0.35	0.42
B1	0.29	0.30	0.16	0.16	0.11
B2	0.13	0.17	0.47	—	0.23
B3	0.06	0.33	—	0.54	0.07
C1	0.10	—	0.26	—	0.64
C2	0.09	0.38	0.28	—	0.26
C3	0.04	0.14	—	0.17	0.64
D1	0.17	0.31	—	0.11	0.41
D2	0.10	0.08	0.16	0.31	0.35
D3	0.09	0.30	—	0.19	0.42

**Table 5:** The collation of the data of the biological prototype scores.

	A1	A2	A3	B1	B2	B3	C1	C2	C3	D1	D2	D3
*Q_j_*	2.85	3.36	4.09	2.37	3.97	2.99	3.24	4.29	3.03	3.25	4.15	2.55

**Table 6:** The orthogonal analysis results.

	A	B	C	D	Result
1	A1	B1	C1	D1	6.09
2	A1	B2	C2	D2	15.26
3	A1	B3	C3	D3	8.43
4	A2	B1	C2	D3	10.21
5	A2	B2	C3	D1	10.36
6	A2	B3	C1	D2	10.75
7	A3	B1	C3	D2	11.27
8	A3	B2	C1	D3	13.84
9	A3	B3	C2	D1	11.63
Mean value 1	9.927	9.190	10.227	9.360	
Mean value 2	10.440	13.153	12.367	12.427	
Mean value 3	12.247	10.270	10.020	10.827	

**Table 7:** The comparison of the scheme before and after improvement.

Structure	Prebionic improvement	After bionic improvement
Upper skewer structure	The regular shape of the upper skewer shaft easily causes the gear skewer to jam and affects the use effect	The groove design that fits the shape of the gear skewer greatly avoids the problem of clamping the skewer
Skewer-pushing structure	The sensitivity of skewer recognition is low, which easily causes the problem of empty skewer threading or nonretraction after threading	The bionic snail eyes design increases the receptors and enhances the sensitivity of the skewer-pushing structure
Walking plate structure	The large-scale die structure is difficult to adapt to all types of threading machines	The bionic Gaboon viper design increases the flexibility of the die plate
Packaging structure	The manual packaging method reduces the efficiency of the whole process	The integration of the packaging structure into the whole machine improves the operation efficiency

## Data Availability

The [Figure and Table] data used to support the findings of this study are included within the article.

## References

[1] Tan R. H., Liu W., Cao G. Z., Shi Y. (2019). Creative design inspired by biological knowledge: technologies and methods. *Frontiers of Mechanical Engineering*.

[2] Huang R. K., Shang L. R., Zhao Y. J. (2021). Biomimic organ architectures and functions by assembling organoid models. *Science Bulletin*.

[3] Li D. P., Ci L. J. (2021). Biomimetics: from biological cells to battery cells. *Science Bulletin*.

[4] Vattm S., Wiltgen B., Helms M., Goel A. K., Yen J. (2010). Dane: fostering creativity in and through biologically inspired design. *Design Creativity*.

[5] Goel A. K., Vattm S., Wiltgen B., Helms M. (2012). Cognitive, collaborative, conceptual and creative—four characteristics of the next generation of knowledge-based CAD systems: a study in biologically inspired design. *Computer-Aided Design*.

[6] Nagel J. K. S., Nagel R. L., Stone R. B., McAdams D. A. (2010). Function-based, biologically inspired concept generation. *Artificial Intelligence for Engineering Design Analysis & Manufacturing*.

[7] Abdala L. N., Fernandes B. R., Ogliari A., Löwer M., Feldhusen J. (2017). Creative contributions of the methods of inventive principles of TRIZ and BioTRIZ to problem solving. *Journal of Mechanical Design*.

[8] Chakrabarti A. (2014). Supporting analogical transfer in biologically inspired design. *Biologically Inspired Design*.

[9] Wilson J. O., Rosen D. Systematic reverse engineering of biological systems. *ASME 2007 International Design Engineering Technical Conferences and Computers and Information in Engineering Conference*.

[10] Shu L. H. (2010). A natural-language approach to biomimetic design. *Ai Edam Artificial Intelligence for Engineering Design Analysis & Manufacturing*.

[11] Vincent J. F., Bogatyreva O. A., Bogatyrev N. R., Bowyer A., Pahl A. K. (2006). Biomimetics: its practice and theory. *Journal of the Royal Society Interface*.

[12] Liu X. M., Wang J. H., Li J. R., Chen L. (2017). The research of product innovation design based on biological prototype. *Chinese Journal of Construction Machinery*.

[13] Cao G. Z., Sun Y. D., Tan R. H., Zhang J., Liu W. (2021). A function-oriented biologically analogical approach for constructing the design concept of smart product in Industry 4.0. *Advanced Engineering Informatics*.

[14] Chen L., Dou H., Wei H. (2020). Product innovation design based on quality function deployment, invention problem solving theory and bionics. *China Mechanical Engineering*.

[15] Liu W., Cao G. Z., Tan R. H., Yu F. (2016). Research on measures to technical realization of multi biological effects. *Journal of Mechanical Engineering*.

[16] Bai Z. H., Mu L., Lin H. C. (2020). Green product design based on the BioTRIZ multi-contradiction resolution method. *Sustainability*.

[17] Ren L. Q., Liang Y. H. (2009). Biocouplers and their coupling methods. *Journal of Jilin University*.

[18] Xu Y. S., Li L. L., Wang C., Zhao Q. K., He J., Wu Y. D. (2019). Application research of coupling bionics in product modeling design. *Mechanical Design*.

[19] Tian L. M., Ke Q. P., Jin E. (2015). Resistance reduction characteristics and mechanism of bionic functional surface coupled with morphology material. *Journal of Agricultural Engineering*.

[20] Hong J., Qian Z. H., Ren L. Q. (2009). Multivariate coupled bionic extension model and its coupled element analysis. *Journal of Jilin University (Engineering Edition)*.

[21] Ren L. Q., Liang Y. H. (2010). Functional characteristics of biological coupling and its realization mode. *Chinese science : technical science*.

[22] Yu Z. P., Xu S. Y., Xiong L., Guang X. L. (2015). Robustness hydraulic pressure control System of Integrated-electro-hydraulic brake system. *Journal of Mechanical Engineering*.

[23] Ren L. Q., Liang Y. H. (2011). Biological coupling generation mechanism. *Journal of Jilin University*.

[24] Ren L. Q., Liang Y. H. (2010). Biological coupling and its taxonomy and characteristics. *Chinese Science*.

[25] Liu S., Zhang Y. Y., Wang Y. N., Zhang Q. J., Li T. (2020). A Method of Root Conflict Problem Solving Based on Bio-Inspired Design. *Mechanical Design and Research*.

[26] Yusof N. S. B., Sapuan S. M., Sultan M. T. H., Jawaid M. (2020). Conceptual design of oil palm fibre reinforced polymer hybrid composite automotive crash box using integrated approach. *Journal of Central South University*.

[27] Chen J. L., Hung S. C. (2017). Eco-innovation by TRIZ and biomimetics design. *2017 International Conference on Applied System Innovation (ICASI)*.

[28] Bogatyrev N. R., Bogatyreva O. A. (2014). BioTRIZ: a win-win methodology for eco-innovation. *Eco-Innovation and the Development of Business Models*.

[29] Ji X., Gu X. J., Dai F., Liu Z. (2014). BioTRIZ-based product innovative design process. *Journal of Zhejiang University*.

[30] Salmaan C., David H., Andrew C., Knott D. (2008). BioTRIZ suggests radiative cooling of buildings can be done passively by changing the structure of roof insulation to let longwave infrared pass. *Journal of Bionic Engineering*.

[31] Liu X. M., Li J. R., Chen L., Chen G. C. (2019). Bionic prototype acquisition incorporating extension and multi-level knowledge modeling. *Journal of Mechanical Engineering*.

[32] Jia L. Z., Liu W., Tan R. H. (2015). Research on product conceptual design method based on biological—technical feature analogy. *Journal of Engineering Design*.

[33] Hou X. T., Liu W., Cao G. Z., Wu Z. F., Guo Z. B. (2017). Research on design method of function combination product based on multi biological effects. *Journal of Engineering Design*.

[34] Zhao W. P., Zhao Z. Y., Lei Y. W., Wang Z. X. (2021). Influencing factor analysis on the seismic behaviors of frame exterior joints based on orthogonal experiments. *Vibration and impact*.

[35] Bai Z. H., Li Y., Song M. J., Huang X. G., He L. (2021). Design and Evaluation of Product Modeling Based on Affordance. *Packaging engineering*.

[36] Feng Z. R., Qin Y. (2021). Characteristic analysis of automobile front longitudinal beam with different sections based on orthogonal test. *Mechanical design and manufacture*.

[37] Hu L. P. (2019). Variable transformation regression analysis (IV)—preference scoring data combination analysis. *Sichuan Mental Health*.

[38] Song Y., Zou P. (2020). AHP analysis of back pressure comfort of military training clothing for college students. *Journal of Liaodong University*.

